# Quantification of Cardiotonic Steroids Potentially Regulated by Paraoxonase 3 in a Rat Model of Chronic Kidney Disease Using UHPLC-Orbitrap-MS

**DOI:** 10.3390/ijms232113565

**Published:** 2022-11-05

**Authors:** Sabitri Lamichhane, Chrysan J. Mohammed, Steven T. Haller, David J. Kennedy, Dragan Isailovic

**Affiliations:** 1 Department of Chemistry and Biochemistry, University of Toledo, Toledo, OH 43606, USA; 2 Department of Medicine, University of Toledo College of Medicine and Life Sciences, Toledo, OH 43614, USA

**Keywords:** endogenous cardiotonic steroids, telocinobufagin, marinobufagin, paraoxonase, urine, SPE, LC–MS

## Abstract

Endogenous cardiotonic steroids (CTSs), such as telocinobufagin (TCB) and marinobufagin (MBG) contain a lactone moiety critical to their binding and signaling through the Na^+^/K^+^-ATPase. Their concentrations elevate in response to sodium intake and under volume-expanded conditions. Paraoxonase 3 (PON3) is an enzyme that can hydrolyze lactone substrates. Here, we examine the role of PON3 in regulating CTS levels in a rat model of chronic kidney diseases (CKD). TCB and MBG were extracted from rat urine samples, and the analyses were carried out using ultra-high pressure liquid chromatography–Orbitrap-mass spectrometry (UHPLC-Orbitrap-MS). Ten-week-old Dahl salt-sensitive wild type (SS-WT) and Dahl salt-sensitive PON3 knockout (SS-PON3 KO) rats were maintained on a high-salt diet (8% NaCl) for 8 weeks to initiate salt-sensitive hypertensive renal disease characteristic of this model. CTS extraction recovery from urine >80% was achieved. For animals maintained on a normal chow diet, the baseline amount of TCB excreted in 24 h urine of SS-PON3 KO rats (6.08 ± 1.47 ng/24 h; or 15.09 ± 3.25 pmol) was significantly higher than for SS-WT rats (1.48 ± 0.69 ng/24 h; or 3.67 ± 1.54 pmol, *p* < 0.05). Similarly, for the same animals, the amount of excreted MBG was higher in the urine of SS-PON3 KO rats (4.74 ± 1.30 ng/24 h versus 1.03 ± 0.25 ng/24 h in SS-WT; or 11.83 ± 2.91 pmol versus 2.57 ± 0.56 pmol in SS-WT, *p* < 0.05). For animals on a high-salt diet, the SS-PON3 KO rats had significantly increased levels of TCB (714.52 ± 79.46 ng/24 h; or 1774.85 ± 175.55 pmol) compared to SS-WT control (343.84 ± 157.54 ng/24 h; or 854.09 ± 350.02 pmol, *p* < 0.05), and comparatively higher levels of MBG were measured for SS-PON3 KO (225.55 ± 82.61 ng/24 h; or 563.19 ± 184.5 pmol) versus SS-WT (157.56 ± 85.53 ng/24 h; or 393.43 ± 191.01 pmol, *p* > 0.05) rats. These findings suggest that the presence and absence of PON3 dramatically affect the level of endogenous CTSs, indicating its potential role in CTS regulation.

## 1. Introduction

**Figure 1:**
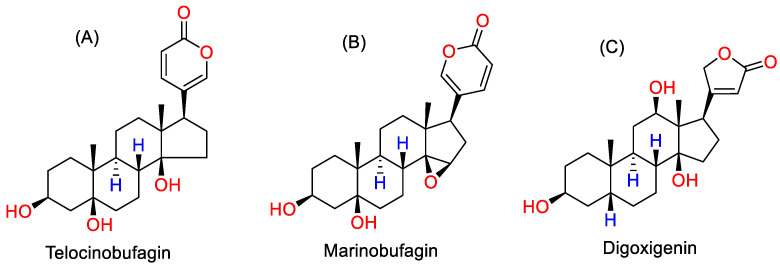
Chemical structures of target CTSs: (**A**) Telocinobufagin (TCB), (**B**) Marinobufagin (MBG) and (**C**) internal standard Digoxigenin (DIG).

Cardiotonic steroids are structurally classified into cardenolides and bufadienolides [1]. These compounds contain a δ-lactone ring (2-pyrone ring) attached to the C-17 position of the steroid nucleus. Bufadienolides such as telocinobufagin (TCB) and marinobufagin (MBG) are characterized by a six-membered unsaturated ring (Figure 1A,B). In contrast, cardenolides, such as ouabain, digoxin, and digoxigenin (DIG) have an unsaturated five-membered ring (Figure 1C). This characteristic ring enables these compounds to bind and either inhibit the ubiquitous transport enzyme Na^+^/K^+^ -ATPase [1] and/or initiate its signaling function [2]. Because of the enzyme inhibitory activity, CTSs are also known as endogenous digitalis-like factors. These compounds are structurally and functionally related to plant cardiac glycosides such as digoxin obtained from *Digitalis purpurea*, which have been extensively used in the treatment of congestive heart failure [3,4]. CTS levels are increased in volume-expanded states, such as CKD, hypertension, and pre-eclampsia [5,6,7,8]. Chronic elevation of CTSs produces deleterious effects, resulting in progressive cardiovascular and renal disease leading to adverse clinical outcomes [2,3]. The Dahl salt-sensitive rat is a well-characterized model of hypertension and kidney injury [9,10]. Herein, this animal model was used to investigate the regulation of CTSs under both basal and salt-loaded conditions.

Paraoxonases (PONs) are a family of endogenous hydrolytic enzymes comprising three isoforms: PON1, PON2, and PON3 [11]. An association between diminished circulating PON lactonase activity and worse clinical outcomes in CKD has been reported in previous studies [12,13,14]. PON3 is the least-studied member of the PON family and much less is known about its physiological substrates. PON3 is synthesized in the liver and released into the blood where it circulates bound to high-density lipoproteins. PON3 gene expression is primarily observed in the liver and kidney, and it is also distributed to various other tissues, including the lung, pancreas, intestine, and eyes [15,16]. While there is some overlap in substrate specificity, PON3 is established primarily as a lactonase. Although some recent studies have established arachidonic acid and its derivatives as the physiological substrates for PON3 [15,17,18], the physiological relevance of their metabolism by PONs remains enigmatic. It has been demonstrated that PON3 hydrolyzes six-membered δ-lactones, which are similar to CTS compounds in their structure [15,17,19,20,21]. Therefore, we sought to determine if PON3 is mechanistically linked to the regulation of CTSs in settings such as CKD. Interestingly, it has been demonstrated that compared to females, increase in PON3 activity in human PON3 transgenic male mice significantly decreases atherosclerotic lesion formation and adiposity in the livers [22].

CTSs are present in mammals, as confirmed by various biochemical [23], immunological [24], and spectroscopic methods [6,25]. These steroid hormones circulate in the blood and are excreted in the urine. Picomolar to nanomolar concentrations of CTSs such as MBG and TCB have been isolated and quantified in human plasma, serum, and urine [2,6,26]. Extraction, detection, and quantification of CTSs in body fluids are critical because these compounds are capable of regulating essential physiological functions, such as sodium homeostasis. Previously used immunoassays are prone to cross-reactivity issues, often leading to poor specificity and high variability at low concentrations. It is, therefore, necessary to develop accurate and precise methods to measure endogenous CTSs at low levels. HPLC coupled with tandem mass spectroscopy (LC-MS/MS) is the analytical method widely used for measuring small biomolecules, such as CTSs at low levels [27,28].

A significant challenge for quantitative LC-MS methods is the suppression or enhancement of the analyte response due to salts and other co-eluting compounds contained in the matrix, known as matrix effects. Biological samples cannot be directly injected into the LC-MS system due to unwanted matrices, leading to various problems such as column clogging and MS signal interferences [29,30]. Therefore, sample preparation is essential before LC separation to minimize matrix effects during ionization and detection of analytes. It also preconcentrates the analyte(s), increasing sensitivity and lowering detection limits. It is often necessary to develop an efficient methodology to extract and recover CTS from urine samples.

Therefore, in the current study, we developed methods for extraction and quantification of CTS using UHPLC-Orbitrap-MS. Using these methods, CTSs were quantified in Dahl salt-sensitive wild type (SS-WT) and PON-3 knock-out (SS-PON3 KO) rats receiving normal chow or high-salt diets for 8 weeks. Our results suggest a potential new role for PON3 in regulating CTS levels in the setting of hypertensive renal disease.

## 2. Results and Discussion

### 2.1. Separation and Measurement of CTS

**Figure 2:**
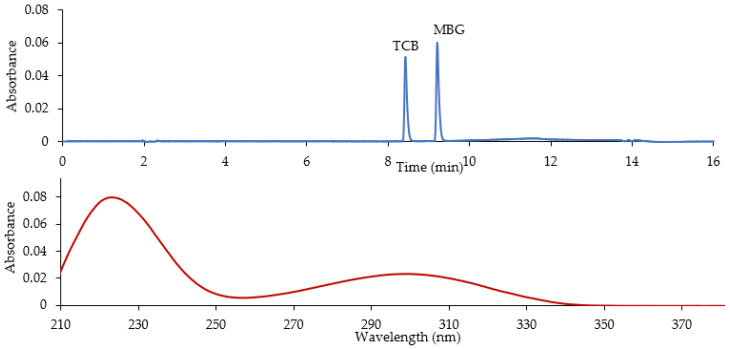
HPLC-PDA profile of CTS dissolved in mobile phase; retention time for TCB and MBG based on UV light absorbance at 300 nm (top figure), UV spectrum showing the absorption maxima for TCB at 223 and 300 nm (bottom figure). MBG shows similar absorption spectrum as TCB.

Initially, the analysis of extracted CTS dissolved in mobile phase was performed using HPLC coupled to photodiode array ((PDA) detector. TCB eluted at 8.42 min, whereas MBG came out at 9.20 min (Figure 2). The corresponding UV spectra showed the characteristic absorption maxima for TCB and MBG at 300 nm and 223 nm [31,32,33], as both absorb UV light at the same wavelengths.

### 2.2. UHPLC-MS Method Optimization

UHPLC-MS has advantages over HPLC-PDA, since it has high resolution and narrow extracted-ion windows, which can then lead to lower detection limit and higher sensitivity. The chromatographic column was selected, and mobile phase, flow rate, and column temperature were optimized to achieve better separation, short retention times, symmetrical peak shapes, and good resolution of CTS. Several LC columns, including Accucore Vanquish C18+, Hypersil GOLD C18 [34], Acquity UPLC BEH C18 [33], and Acquity HSS T3 C18 [35], were investigated in this study. An Acquity HSS T3 column was selected for LC-MS analyses.

Since TCB and MBG have closely related structures, their separation was primarily dependent on the mobile phase composition. Several trials were performed with various proportions of gradient elution systems of methanol–water, acetonitrile–water, and formic acid in both solvents. These compounds eluted very closely when using methanol as an organic phase solvent. Acetonitrile was better than methanol as it provided lower column pressure and produced better separation and resolution. The mobile phase consisting of water with 0.1% formic acid (A) and acetonitrile with 0.1% formic acid (B) was chosen because it allowed the best separation for these analytes in the samples and yielded a good peak shape. Using these optimized conditions, the chemical profiles of urine samples were acquired.

**Figure 3:**
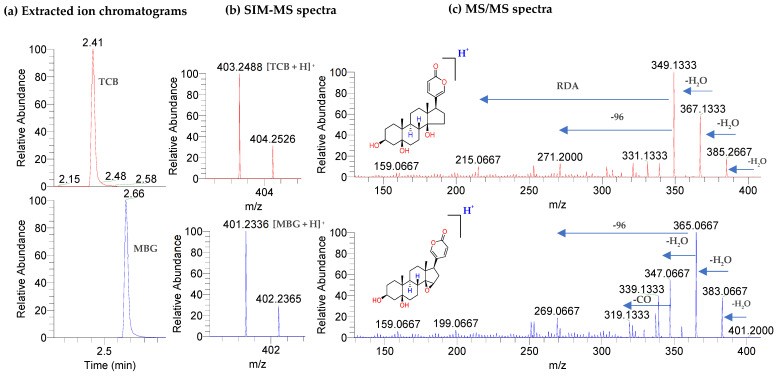
Analysis of CTS spiked in mobile phase by UHPLC-Orbitrap-MS and MS/MS: (**a**) EICs showing retention times of TCB and MBG; (**b**) SIM scan mass spectra, showing protonated TCB [M + H]^+^ = *m/z* 403.2488, and MBG [M + H]^+^ = 401.2336; (**c**) MS/MS spectra for the [M + H]^+^ ions at *m/z* values of 403.2488 and 401.2336.

The UHPLC-Orbitrap-ESI-MS method was applied to separate and detect standard CTSs spiked in solvent and CTSs extracted from urine. The retention times and mass spectra of urine CTSs were equivalent to standard CTSs (Figure 3), which confirmed their identities.

Baseline separation of CTS was achieved in 3 min using UHPLC, with retention times of 2.41 min (TCB) and 2.66 min (MBG) min. The base peaks in the ESI-MS spectra correspond to protonated TCB (exact *m/z* 403.2479) and MBG (exact *m/z* 401.2323) with *m/z* values of 403.2488 and 401.2336, respectively. MS/MS spectra of TCB and MBG in Figure 3 shows fragment ions produced by consecutive losses of water molecules, such as [M + H-H_2_O]^+^, [M + H−2H_2_O]^+^, [M + H−3H_2_O]^+^, and [M + H−4H_2_O]^+^, which are consistent with previously published results [6,36]. Fragment with *m/z* 349.1333 was the base peak in MS/MS spectrum of TCB and fragment with *m/z* 365.0667 was the base peak in MS/MS spectrum of MBG. From *m/z* 50 to 250, steroid fragmentation resulted from different rearrangement reactions, such as retro-Diels Alder (RDA) [36]. Protonated DIG with *m/z* 391.2481 (exact *m/z* of 391.2493) eluted at 1.13 min (Supplementary Figure S1). The calculated mass accuracies for these ions were <4 ppm (Supplementary Table S1).

### 2.3. Extraction of CTS from Aqueous Solution

Given their relevance to both physiologic (sodium homeostasis) and pathophysiologic (cardiovascular inflammation, fibrosis, etc.) processes, the measurement of CTSs in biofluids using sensitive and specific analytical methods is necessary for determining their role in health and disease. Since urine presents challenges to sample analysis given its complexity as a biological matrix, special attention should be taken during sample preparation. Optimizing sample preparation procedures is vital to minimize interferences from the sample matrix in LC-MS analyses and achieving satisfactory recovery of the analytes.

Parameters such as the type of SPE cartridge, loading and elution volumes, eluent solvent, and flow rate were optimized, as they can significantly affect extraction efficiency. In the current study, a range of solvents with different polarities, including dichloromethane, ethyl acetate, acetone, methanol, and acetonitrile, was tested.

**Figure 4:**
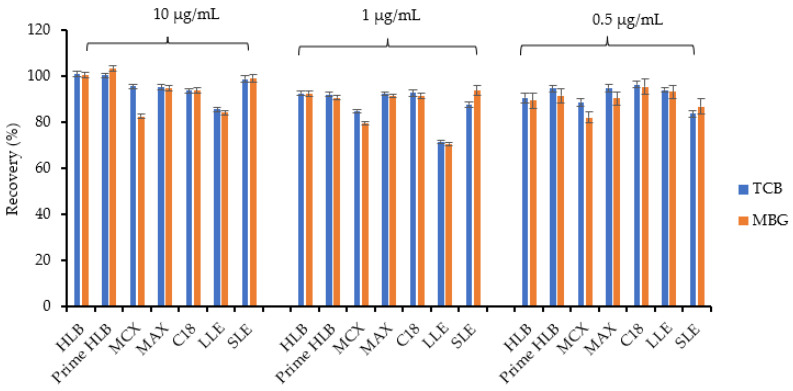
Percent recoveries of CTSs spiked in H_2_O determined by HPLC-PDA after extraction with various extraction methods. The HPLC-PDA analyses were carried out in triplicate (*n* = 3).

Liquid–liquid extraction (LLE) [37], and solid-phase extraction (SPE) cartridges such as C18 [38,39], Mixed-mode Cation eXchange (MCX) [40], and Hydrophilic-Lipophilic Balance (HLB) [41,42], have been previously applied for the extraction of bufadienolides from different matrices. Extraction recoveries of 10, 1, and 0.5 µg/mL TCB and MBG mixture spiked in H_2_O were determined by comparison of absorbance of CTS before and after SPE (Supplementary Equation (S1)) using various cartridges and HPLC-PDA detector (Figure 4).

For a mixture of 10 µg/mL and 1 µg/mL, and 0.5 µg/mL of CTS spiked in H_2_O, recovery was higher for ISOLUTE_TM _ Supported Liquid Extraction (SLE), HLB, and Prime HLB cartridges as determined by HPLC-PDA. LLE showed limited extraction efficiency of the analyte. MCX and Mixed-mode Anion eXchange (MAX) cartridges did not produce clean spectra that were free of other contaminant peaks.

### 2.4. Extraction Recovery of CTS from Urine

**Figure 5:**
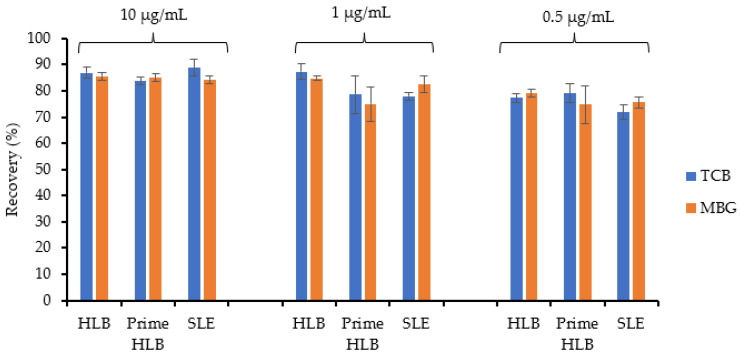
Percent recoveries of CTS spiked in urine after extraction with HLB, Prime HLB and SLE cartridges determined by HPLC-PDA. The analyses were carried out in triplicate (*n* = 3).

Since HLB, Prime HLB, and SLE performed well when CTS was spiked in water, these cartridges were again tested for CTS spiked in normal chow urine at the same three concentrations (Figure 5).

**Figure 6:**
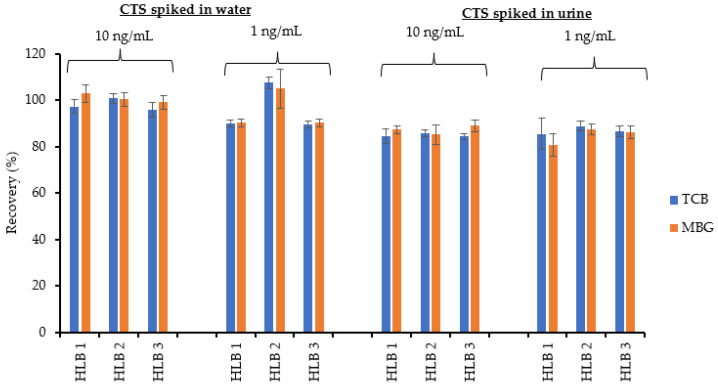
Percent recoveries of CTS spiked in water and urine at concentrations of 10 ng/mL and 1 ng/mL determined by UHPLC-MS after extraction with HLB cartridges. The analyses were carried in triplicates (*n* = 3).

Overall, HLB had the highest recovery compared to the other two cartridges. It was observed that the CTS recovery decreased for urine spiked with lower concentrations of CTSs vs. those spiked with higher concentration of CTSs. CTSs were successfully extracted from rat urine samples using HLB cartridges with recoveries >80%. The best recoveries from SPE were achieved when washing was carried out with 5% methanol, and elution with 100% methanol. HLB was chosen for the extraction of CTS from rat urine since it yielded higher recovery, produced consistent and reproducible results, and the extraction procedure was simple and efficient. After figuring out that HLB was a suitable extraction cartridge for maximum CTS recovery from urine, it was tested for reproducibility using three different cartridges at lower concentrations (1 and 10 ng/mL) (Figure 6).

Similar extraction experiments were carried out for CTS spiked separately in water and urine samples. The extraction recovery was calculated by comparing the mean peak areas in monoisotopic peaks of pre-extraction spiked matrix samples with post-spiked urine samples (Supplementary Equation (S1)).

The recovery was higher for CTS spiked in water (>89%) than spiked in urine (>80%) due to the difference in the matrix effects between these two samples.

### 2.5. Matrix Effects and Internal Standard Evaluation

A co-eluted matrix can alter the ionization efficiency and influence the detection and quantification of an analyte in urine samples. Finding urine blanks that did not contain CTSs was not possible as unknown amounts of these compounds are always present. Because of these reasons, quantifying endogenous CTSs in urine is challenging as constructing a calibration curve by spiking internal standard in a blank solution may not be optimal. Previously, urine and plasma were stripped of steroids by charcoal and used as blanks for the quantification of endogenous steroids [43]. The present study followed a similar method to remove endogenous CTSs from urine samples. However, not only the endogenous steroids but also the other matrices were removed, resulting in a control that differed too significantly in the matrix from the actual urine samples. Therefore, this method could not be used for quantification purposes.

The matrix effects were assessed by comparing the mean peak areas of DIG standard spiked after SPE with the mean peak areas of DIG spiked into solvent. DIG was spiked into NC and HS urine before and after SPE at 1 ng/mL, 5 ng/mL, and 10 ng/mL concentrations to obtain the recovery percentage from urine and facilitate internal standard selection. SPE was carried out, and the chromatographic peak areas measured by UHPLC-MS were compared between different matrices (Table 1).

The recoveries of digoxigenin at three different concentrations spiked in urine were calculated using Supplementary Equation (S1) to ensure SPE worked for DIG as well as for other CTSs. There was no significant difference in the peak area values for DIG spiked in urine before and after SPE. The recovery was >75% for normal chow (NC) urine and >80% for high-salt (HS) urine, meaning that not much analyte was lost during extraction. However, there were significant differences in the peak area values between NC and HS samples for the same concentration of DIG spiked in them. This indicates that DIG suffers from ionization suppression during ESI-MS of NC samples.

The matrix effects were calculated using Supplementary Equation (S2) for both NC and HS urine. A huge variability was observed; for NC urine, the matrix effect was more than 50% for all three concentrations of DIG, whereas for HS urine, the matrix effect was around 10% or less. This indicates that DIG in NC samples suffers from a matrix effect. Therefore, spiking DIG into the same blank (negative control urine) and constructing a calibration curve for the quantification of CTS in NC and HS urine samples does not give accurate results. Therefore, the method of standard addition calibration was used to compensate for these matrix signal suppressions in NC rats and an internal standard calibration was used for HS urine samples. Standard addition calibration curves for quantification of TCB and MBG in normal chow rat urine are shown in Supplementary Figures S2 and S3. Internal standard calibration curves to quantify TCB and MBG in HS urine are shown in Supplementary Figure S4.

### 2.6. Application of the Developed Methods for the Extraction and Quantification of CTS from Rat Urine Samples

SS-WT (control) and SS-PON3 knockout (KO) rats were maintained on a high-salt diet (8% NaCl) to initiate CKD. The 24 h urine volume for rats maintained on the NC diet was 7.70 ± 1.57 mL and the HS diet was 79.90 ± 8.77 mL. The role of PON3 in regulating the CTS levels in these well-characterized models of high-salt-induced CKD was examined. Five representative animal samples (*n* = 5) were taken from each model and CTSs were quantified. TCB and MBG were successfully extracted from the rat urine samples and quantified using UHPLC-Orbitrap MS. As mentioned above, standard addition and internal standard methods were respectively used for the quantification of CTS in NC and HS urine samples (Table 2). The developed SPE and UHPLC-MS method could be optimized and applied for the potential extraction and quantification of cardenolides such as ouabain. Ouabain is less hydrophobic than TCB and MBG because it consists of a sugar moiety (L-rhamnose attached to it on 3β-OH position) and is expected to elute earlier than the other CTS from the reversed-phase UHPLC column.

The presence of endogenous CTSs in a picomolar-to-nanomolar concentration range and the dramatic increase in plasma and urine CTS levels in clinical and experimental volume expanded states has been reported by several studies [5,6,7,27,44,45,46,47], which are comparable to the concentrations of CTSs measured in the present study.

From Table 2, the results clearly show that the quantity of TCB and MBG excreted in the 24 h urine volume of SS-PON3 KO rats were significantly higher than SS-WT in the urine of rats on normal chow diet. Following 8 weeks of a high-salt diet, the SS-PON3 KO rats had significantly increased levels of TCB compared to SS-WT control as well as a trend of higher levels of MBG in urine of SS-PON3 KO rats versus SS-WT rats. This suggests the potential involvement of PON3 in the regulation of CTS, probably via its lactonase activity.

It is evident from Table 2 that after 8 weeks of HS treatment, both SS-WT and SS-PON3 KO animals show a significant increase in CTS levels, compared to their respective controls (NC rats). Many previous studies have reported higher plasma CTS levels in CKD patients compared to healthy individuals [48,49]. In a study on the human population, the mean concentration of TCB in healthy volunteers (1.80 ng/mL) was significantly lower than that in patients with chronic renal failure (6.86 ng/mL) [11,18]. Fedorova et al. investigated the possible role of MBG in hypertension in Dahl salt-sensitive rats on a high NaCl intake. They found a fourfold increase in the renal excretion of MBG, 38.9 ± 7.6 pmol, versus baseline, 9.1 ± 1.3 pmol (*p* < 0.01) [50].

Our findings suggest that the endogenous TCB concentration was higher than MBG in all samples, which is in agreement with data from other studies [6] and consistent with reports that TCB has one of the most potent activities among all CTSs [2,51,52,53]. Additionally, the urine of SS-PON3 KO rats contained higher CTS levels than urine from SS-WT rats for both control and high-salt samples, suggesting its role in CTS regulation. Our study is the first to investigate the potential role of PON3 in the regulation of CTSs. However, further studies are necessary to determine the mechanisms by which PON3 regulates these steroid compounds. To gain a better understanding of the physiological role of PON3 in animals, in vivo studies are in progress [54,55,56], and associated manuscripts will be submitted for publication.

## 3. Materials and Methods

### 3.1. Reagents

LC-MS and HPLC-grade acetonitrile, methanol, water, and formic acid were purchased from Fisher Scientific (Pittsburgh, PA, USA). TCB (Figure 1A) standard was obtained from Baoji Herbest Bio-Tech Company, Baoji, China and MBG (Figure 1B) was from Cayman Chemicals, Ann Arbor, MI, USA. Digoxigenin (Figure 1C), used as the internal standard (IS), is a certified reference standard purchased from Millipore sigma, Milwaukee, WI, USA. Negative control urine was purchased from Supelco, Round Rock, TX, USA.

### 3.2. Materials and Instruments

The cartridges used for the SPE were as follows: HLB, MCX, MAX and Sep-Pak C18 Plus Light SPE (Waters, Milford, MA, USA). SLE (Biotage, Charlotte, NC.) was also tested. Two-mL clear glass vials and glass inserts were acquired from Sigma (St. Louis, MO, USA). Two-mL centrifuge tubes were acquired from Fisher Scientific (Pittsburgh, PA, USA). Syringes (3 and 10 mL) were purchased from Becton, Dickinson and Company (Franklin Lakes, NJ, US). The heated vacuum concentrator was from Eppendorf (Hamburg, Germany).

Two different instrumentation systems were used for the study. Initial work was performed using a Shimadzu (Addison, IL, USA) HPLC instrument comprised of a CBM-20A system controller, two LC-20AD pumps, a DGU-20A3 degasser, a SIL-20A HT autosampler, and an SPD-M20A PDA detector. Chromatographic separations were achieved using a Zorbax SB C18 column (150 × 4.6 mm, 3.5μm) equipped with a Zorbax SB C18 guard column (12.5 × 4.6 mm, 5 μm) from Agilent Technologies, Palo Alto, CA, USA. Further experiments were performed using a Thermo Vanquish Flex ultra-high-performance liquid chromatography (San Jose, CA, USA). The chromatographic column was a Waters (Waters, Milford, MA, USA) Acquity HSS T3 (100 × 2.1 mm, 1.8 μm) preceded by a Van Guard pre-HSS T3 column (5 × 2.1 mm, 1.8 μm). UHPLC was connected to a Thermo Orbitrap Fusion Tribrid mass spectrometer equipped with a heated electrospray ionization (ESI) source used for ionization and detection for the detection and quantification of CTSs. Data acquisition and quantification were performed using Xcalibur software, version 2.1 (Thermo Scientific, Waltham, MA, USA).

### 3.3. Methods

#### 3.3.1. Standard Solution Preparation

The samples were solubilized in 1 mL of methanol to create stock solutions used to develop the analytical method. The working solutions were freshly prepared by diluting standard solutions with methanol at appropriate concentrations. All stock standards and LC-MS standards were stored at −20 °C and allowed to equilibrate at room temperature for at least 15 min before use. The standard stock concentrations for CTS were further checked using a Hewlett-Packard HP 8452A UV-Vis spectrophotometer (Hewlett-Packard, Palo Alto, CA, USA) and the literature [57] extinction coefficient.

All the standards were diluted to 0.5, 1, and 10 μg/mL concentrations for CTS recovery experiments performed using HPLC-PDA. Similarly, for LC-MS/MS recovery experiments, the stock solution containing a mixture of TCB and MBG with the final concentration of 100 ng/mL was prepared in methanol and further diluted to 1 ng/mL and 10 ng/mL with UHPLC mobile phase. For standard addition calibration, the TCB and MBG mixtures were spiked into the urine at concentrations ranging from 0.10 ng/mL to 10 ng/mL. For internal standard calibration, 100 µL of urine were spiked with two CTSs containing 5 µL of 100 ng/mL of internal standard (DIG) at concentrations ranging from 0.05 ng/mL to 5.0 ng/mL.

#### 3.3.2. HPLC-PDA Method Development

Initial experiments were performed using an HPLC-PDA instrument. The solvent flow rate was 1 mL/min, and the sample injection volume was 20 μL. Mobile phase A consisted of water containing 0.1% formic acid (FA), while mobile phase B was acetonitrile containing 0.1% FA. The column was equilibrated with 30% mobile phase A and 70% mobile phase B. The gradient that was used for the separation of CTS was 0.1–3 min 30% of B, 3–9 min 95% of B, 9–11 min 95% of B, 12–16 min 30% of B, and the run was stopped at 16 min. This gradient elution allowed the efficient separation of the CTS and interfering components in the urine. LC-PDA data were acquired using Xcalibur software version 2.1 (Thermo Scientific, Waltham, MA, USA). The chromatographic peak areas of TCB and MBG measured at 300 nm were used to calculate extraction recovery. This analytical approach was used for preliminary evaluation of the extraction efficiency for CTS using various extraction methods.

#### 3.3.3. UHPLC-MS Method Development

UHPLC-MS gradient methods were developed for detecting and quantifying low abundance CTS in urine samples based on the HPLC-PDA methods created. Orbitrap Fusion MS consists of three mass analyzers: a quadrupole, a linear ion trap, and an Orbitrap.

The mobile phases were water with 0.1% FA (A) and acetonitrile with 0.1% formic acid (B). The column was equilibrated with 30% B for 15 min prior to the initial injection. The gradient started with 30% B and was increased to 95% B in 5 min (curve = 5). The gradient was held at 95% B until 6.5 min, then decreased to 30% B at 7 min (curve = 5) and maintained at 30% B for 5 min to re-equilibrate the column. The column temperature was optimized to 45 °C with the flow rate at 0.5 mL/min, and gradient elution was performed with a total run time of 12 min. The sample injection volume was 20 μL.

The ESI capillary spray voltage was 3000 V, the vaporizer temperature was 285 °C, and the ion transfer tube temperature was 325 °C. Nitrogen was the sheath (40 arbitrary units), auxiliary (10 arbitrary units), and sweep (1 arbitrary units) gas. ESI with selected-ion monitoring (SIM)-MS in positive ion mode was used to quantify CTS. MS and MS/MS were performed simultaneously, and collision-induced dissociation (CID) was used to fragment CTS precursor ions. Parent ions were detected using the Orbitrap mass analyzer and fragment ions were analyzed with a linear ion trap mass analyzer. SIM channels that were monitored for quantification corresponded to the protonated ([M + H]^+^) ions of TCB, MBG and DIG with mass-to-charge (*m/z*) ratios of 403.25, 401.23, and 391.25, respectively. All samples were analyzed by LC-MS in triplicate. The extracted ion chromatogram (EIC) peak areas of monoisotopic CTS ions were used for quantification.

### 3.4. Animals

**Figure 7:**
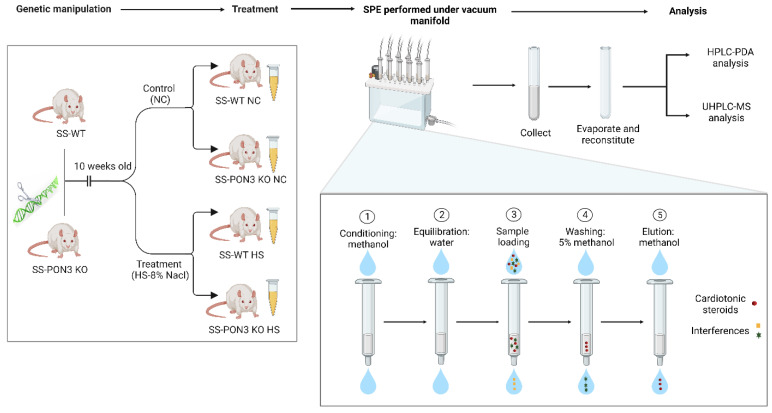
Animal treatment, urine collection and sample preparation using SPE before injection into the analytical instruments. (Created with BioRender.com) Date when last assessed: 16 September 2022.

All animal experiments were performed in compliance with the National Institutes of Health (NIH) Guide for the Care and Use of Laboratory Animals under protocols approved by the University of Toledo Institutional Animal Care and Use Committee. Dahl salt-sensitive rats (SS^Mcwi^, herein referred to as SS rats) and PON3 knock-out animals (designated SS-Pon3^em1Mcwi^, herein referred to SS-PON3 KO rats) were used for the following studies as outlined in Figure 7.

SS-PON3 KO rats were created at the Medical College of Wisconsin Gene Editing Rat Resource Center by injecting a CRISPR targeting the sequence ATCATGATGGGTTCGACCAGG into SS rat embryos, resulting in a 16 bp frameshift deletion in exon 4. The founder animals were genotyped by the Cel-1 assay and confirmed by Sanger sequencing. The founders were then backcrossed to the parental strain and subsequent litters were genotyped by fluorescent genotyping. Ten-week-old, aged-matched male rats were maintained on a high-salt diet (8% NaCl, Envigo, Teklad diets, Madison, WI) for 8 weeks to initiate salt-sensitive hypertensive renal disease that is characteristic of this model, while aged and sex-matched control animals were maintained on a control diet (designated “NC”; 0.3% NaCl, Envigo, Teklad diets, Madison, WI; Figure 7). The genotype of all animals enrolled in the study protocol was confirmed by DNA sequencing.

### 3.5. Comparison of Sample Preparation Techniques

LLE, SPE, and SLE were tested for the extraction of CTS from aqueous solution as well as from urine samples to determine which cartridge generated the cleanest extract and the highest recovery. LLE was performed using the aqueous phase (water) and organic phase (chloroform, ethyl acetate, and hexane). SLE experiments were carried out using ISOLUTE^TM^ SLE cartridges. Aqueous sample was loaded into the cartridge, left for 5 min, and then the analytes were eluted twice with 700 μL of dichloromethane/isopropyl alcohol (95/5, *v*/*v*). Various commercially available SPE cartridges (C18, HLB, prime HLB, MCX, and MAX) were investigated for efficient CTS recovery. The eluates from these SPE cartridges were collected and analyzed with HPLC-PDA and UHPLC-ESI-MS.

### 3.6. Urine Sample Preparation (SPE Protocol)

The 24 h urine samples were collected from SS-WT and SS-PON3-KO rats on normal chow (NC) and high-salt diets (HS). Samples were centrifuged at 10,000 rpm for 10 min at 4˚C; the supernatants were transferred to different vials and stored frozen. For analysis, urine samples were taken and ice-thawed at room temperature. A total of 150 µL of urine was transferred to a 1 mL tube, to which 850 µL H_2_O was added. SPE was performed using HLB cartridges as shown in Figure 7 to extract CTS.

Before loading urine samples, the cartridges were conditioned with 1 mL of methanol followed by 1 mL of water. An aliquot of 1 mL of urine was loaded onto the SPE extraction cartridge. Next, 1 mL of 5% methanol solution was used for cartridge wash, and then CTSs were eluted with 1 mL of methanol. Finally, the eluate was evaporated to dryness at 30 °C for 40 min (Refrigerated Centrivap Concentrator, Labconco^®^), and the residue was reconstituted in 150 μL of 30:70 acetonitrile: water (*v*/*v*) containing 0.1% FA for LC-MS and LC-PDA analyses. A 20 μL aliquot of the supernatant was injected into the UHPLC-MS systems for analysis.

### 3.7. Quantification of CTS in Rat Urine Samples

A standard addition method was developed to quantify CTS in normal chow rat urine. A concentration series of CTS (0.10 ng/mL to 10 ng/mL) was prepared, spiked into urine, subjected to SPE and measured by UHPLC-MS. The equation of the calibration curve determined using Microsoft Excel was used to calculate the endogenous CTS concentration, which was also confirmed by extrapolation of calibration curve to the *x*-axis. Internal standard calibration method was developed to quantify CTS in high-salt-diet rats. Digoxigenin (Figure 1C), a cardenolide compound structurally similar to TCB and MBG and not found endogenously in rat urine, was used as an internal standard. A 5 µL of 100 ng/mL DIG was added to the calibration solutions containing from 0.05 ng/mL to 5.0 ng/mL of CTS and the unknown urine samples. The ratios of CTS and DIG peak areas were determined and the calibration curve was constructed. Negative control urine, a synthetic urine, was verified for the absence of CTS using UHPLC-MS and used as a blank matrix for the preparation of calibration curves.

### 3.8. Statistical Analysis

Microsoft Excel was used to present the data as the mean ± standard deviation of the mean, and to prepare graphs. For comparisons between two groups, the Mann–Whitney U test was applied using R software, version 4.2.1. A *p*-value < 0.05 was considered as statistical significance.

## 4. Conclusions

CTSs were extracted and quantified in rat urine samples using a UHPLC-MS method. Although previous studies have examined PON’s potential role in various diseases, to the best of our knowledge, this is the first study to examine the involvement of PON3 on CTS regulation in a well-characterized model of CKD. Interestingly, our findings suggest that there is a potential relationship between PON3 and high-salt-induced CKD in SS rats. This suggests that CTSs may be a putative substrate for PON3, although additional in vitro and in vivo studies are needed to identify a potential mechanistic link between PON3 and endogenous CTS whereby the lactonase activities of PON3 hydrolyze CTS, thus establishing CTSs as one of the novel substrates for PON3.

The developed method provides a sensitive and specific tool which can be used to detect and monitor CTS metabolism both clinically and experimentally. As such, this work represents a significant advance in our ability to understand the role of both CTSs and PON3 in health and disease.

## Figures and Tables

**Table 1:** Calculation of DIG recovery and matrix effects from normal chow urine samples.

Urine Sample	10 ng/mL		5 ng/mL		1 ng/mL	
	Recovery (%)	RSD (%)	Recovery (%)	RSD (%)	Recovery (%)	RSD (%)
Normal chow (*n* = 3)	90.16	5.36	78.32	6.64	76.10	3.34
High-salt (*n* = 3)	95.37	3.97	89.36	1.39	82.54	3.03
	Matrix effect (%)	RSD (%)	Matrix effect (%)	RSD (%)	Matrix effect (%)	RSD (%)
Normal chow (*n* = 3)	55.68	0.03	61.56	0.02	67.83	0.23
High-salt (*n* = 3)	6.47	0.02	9.06	0.01	10.09	0.04

**Table 2:** The comparison of 24 h concentration levels of TCB and MBG in rat urine based on the presence and absence of PON3 (calculated by Mann–Whitney U test, *n* = 5).

Group	CTS	Amount Excreted in 24 h (pmol) (Mean ± SD)		*p*-Value
		SS-WT	SS-PON3 KO	
NC (normal chow) (*n* = 5)	TCB	3.67 ± 1.54	15.09 ± 3.25	0.007937
	MBG	2.57 ± 0.56	11.83 ± 2.91	0.007937
HS (high salt) (*n* = 5)	TCB	854.09 ± 350.02	1774.85 ± 175.55	0.007937
	MBG	393.43 ± 191.01	563.19 ± 184.5	0.1508

## Data Availability

The datasets generated and/or analyzed during the current study are available from the corresponding author on reasonable request.

## Supplementary Materials

The supporting information can be downloaded at: https://www.mdpi.com/article/10.3390/ijms232113565/s1.

## Abbreviations

CTS	cardiotonic steroids
PON	paraoxonase
CKD	chronic kidney disease
TCB	telocinobufagin
MBG	marinobufagin
DIG	digoxigenin
WT	wild type
KO	knockout
SS	salt-sensitive
NaCl	sodium chloride
SPE	solid-phase extraction
HLB	hydrophilic-lipophilic balanced
UHPLC	ultra-high pressure liquid chromatography
MS	mass spectrometry
PDA	photodiode array
