# Supplementary material for: Quantification of Cardiotonic Steroids Potentially Regulated by Paraoxonase 3 in a Rat Model of Chronic Kidney Disease Using UHPLC-Orbitrap-MS
Source: Int J Mol Sci. 2022 Nov 5;23(21):13565. doi: 10.3390/ijms232113565 (PMC9654389; doi:10.3390/ijms232113565)
Supplement: Supplementary file 1 [file ijms-23-13565-s001.zip › ijms-1956422-supplementary.pdf]

Supplementary materials

Quantification of Cardiotonic Steroids Potentially Regulated by Paraoxonase 3 in a Rat Model of Chronic Kidney Disease using UHPLC-Orbitrap-MS

*Sabitri Lamichhane [1], Chrysan J. Mohammed [2], Steven T. Haller [2], David J. Kennedy [*2], Dragan Isailovic [*1]*

[1]Department of Chemistry and Biochemistry, University of Toledo, Toledo, OH 43606,

[2]Department of Medicine, University of Toledo Medical Center, Toledo, OH 43614

Equation S1. Extraction recovery calculation

The extraction recovery of an analyte from an aqueous sample or urine is calculated using the equation given below

$$Extraction\ recovery = \frac{A}{B} \times 100\% \ldots\ldots\ldots (1)$$

A: Peak area of analyte spiked before extraction

B: Peak area of analyte spiked in water (for aqueous sample)

Peak area of analyte spiked after extraction (for urine sample)

Equation S2. Matrix effect calculation

The matrix effect for digoxigenin is calculated using the following equation

$$Matrix\ effect = \frac{A-B}{A} \times 100\% \ldots\ldots\ldots (2)$$

A: Peak area of analyte in standard solution

B: Peak area of analyte in urine which has undergone SPE and later spiked with analyte with same concentration as standard sample

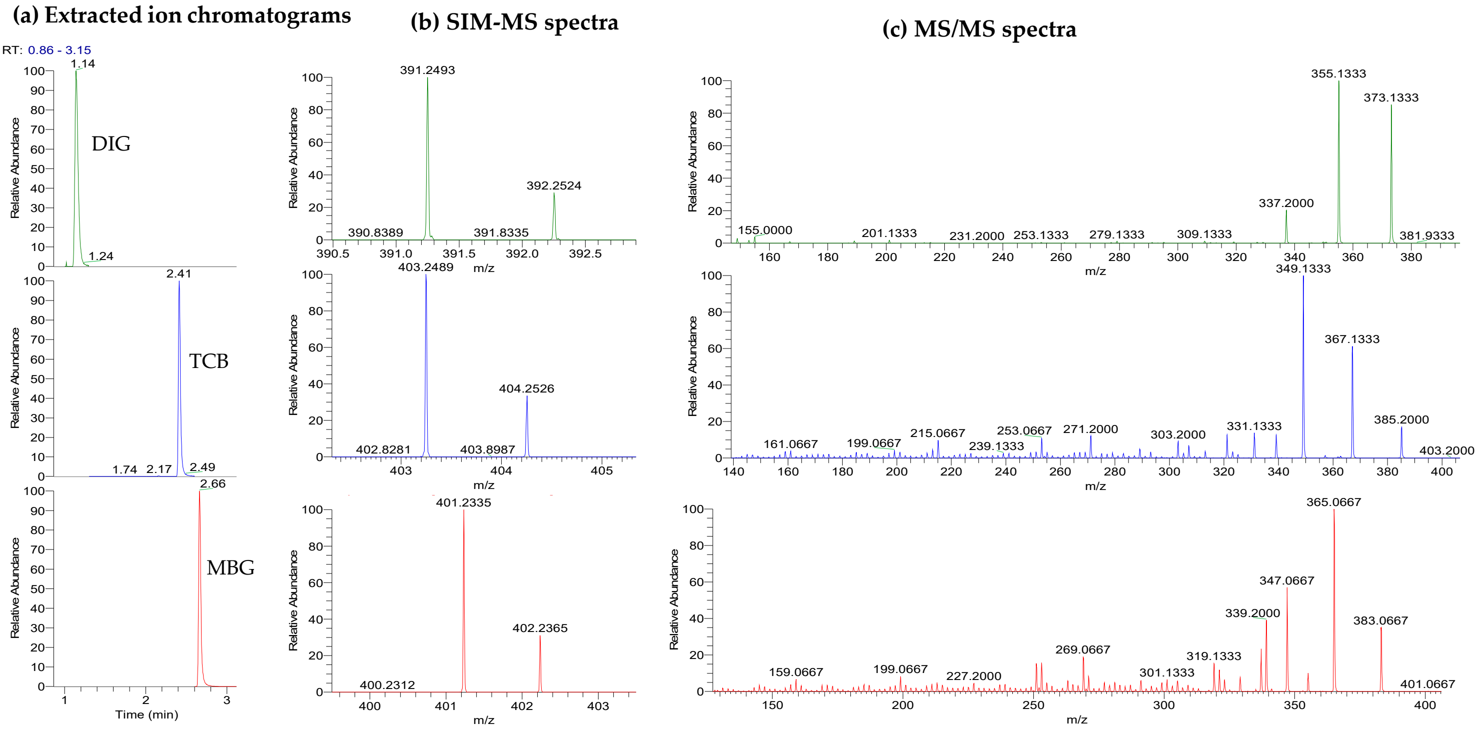

Figure S1. Analysis of CTS and DIG by UHPLC-Orbitrap-MS/MS: (a) Extracted-ion chromatograms showing retention times of DIG, TCB and MBG; (b) SIM scan mass spectra, showing protonated DIG, TCB and MBG ions (c) MS/MS spectra of $[M + H]^+$ monoisotopic precursor ions with *m*/*z* values of 391.2493, 403.2489 and 401.2336.

Table S1. Mass accuracy calculation

CTS compound	Theoretical *m/z*	Experimental *m/z*	Mass accuracy (ppm)
TCB	403.2479	403.2489	2.5
MBG	401.2323	401.2335	3.2
DIG	391.2479	391.2493	3.6

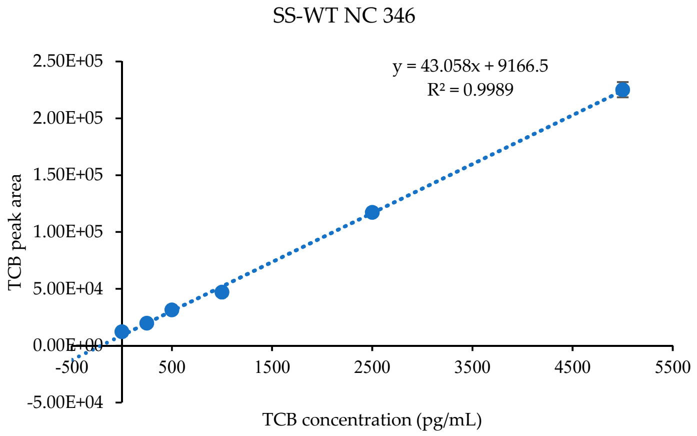
SS-WT NC 346
y = 43.058x + 9166.5
R² = 0.9989
TCB peak area
TCB concentration (pg/mL)

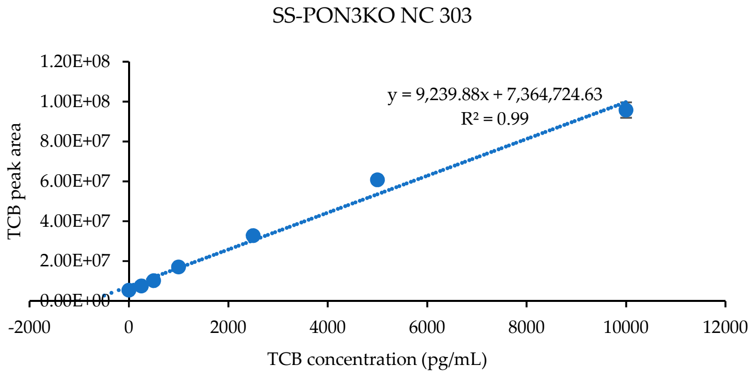
SS-PON3KO NC 303
y = 9,239.88x + 7,364,724.63
R² = 0.99
TCB peak area
TCB concentration (pg/mL)

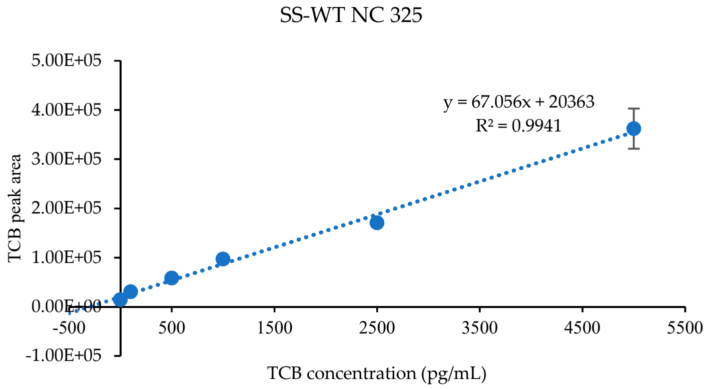
SS-WT NC 325
y = 67.056x + 20363
R² = 0.9941
TCB peak area
TCB concentration (pg/mL)

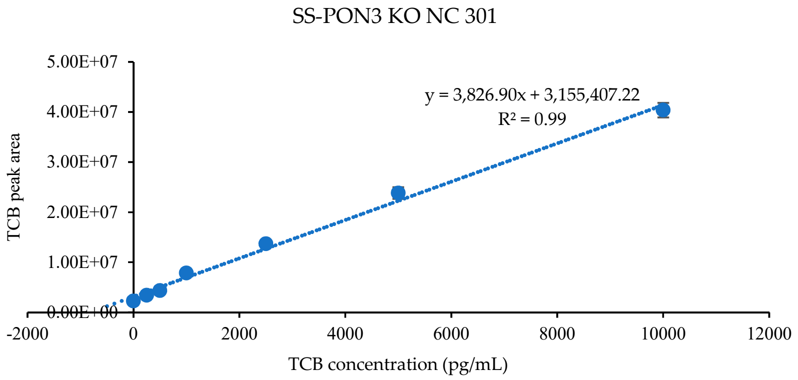
SS-PON3 KO NC 301
y = 3,826.90x + 3,155,407.22
R² = 0.99
TCB peak area
TCB concentration (pg/mL)

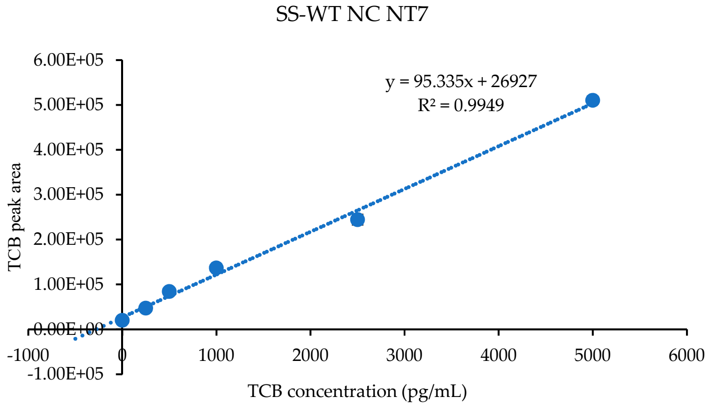
SS-WT NC NT7
y = 95.335x + 26927
R² = 0.9949
TCB peak area
TCB concentration (pg/mL)

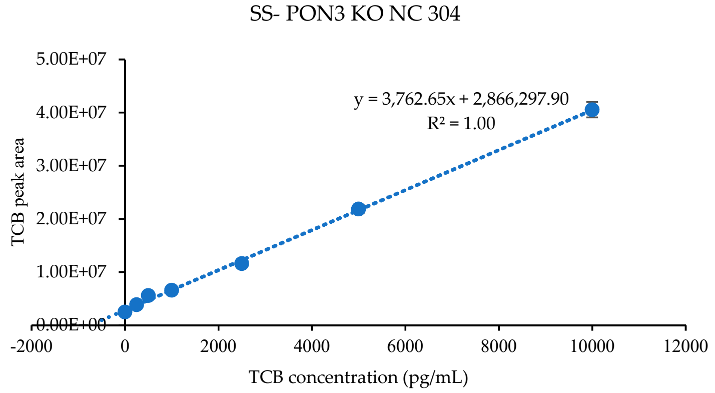
SS- PON3 KO NC 304
y = 3,762.65x + 2,866,297.90
R² = 1.00
TCB peak area
TCB concentration (pg/mL)

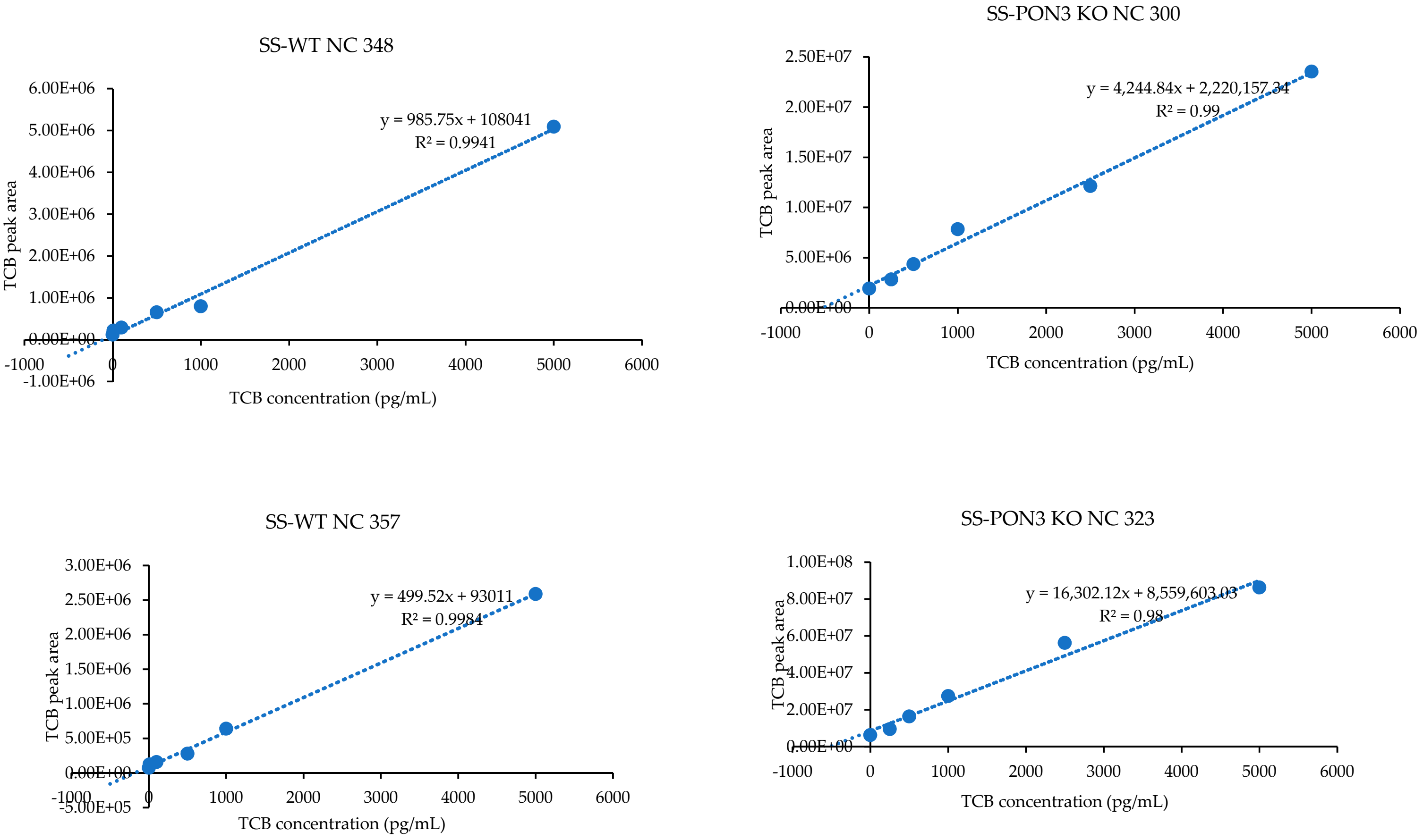

Figure S2. Standard addition calibration curves for quantification of TCB in salt-sensitive wild-type (SS-WT) and paraoxonase 3 knockout (SS-PON 3 KO) rat urine samples on a normal chow diet.

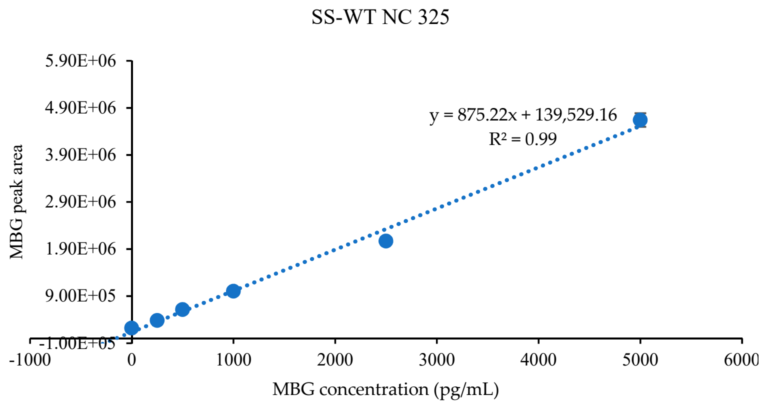
SS-WT NC 325
y = 875.22x + 139,529.16
R² = 0.99
MBG peak area
MBG concentration (pg/mL)

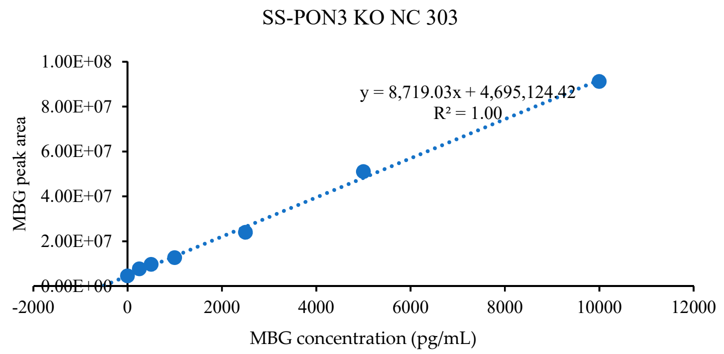
SS-PON3 KO NC 303
y = 8,719.03x + 4,695,124.42
R² = 1.00
MBG peak area
MBG concentration (pg/mL)

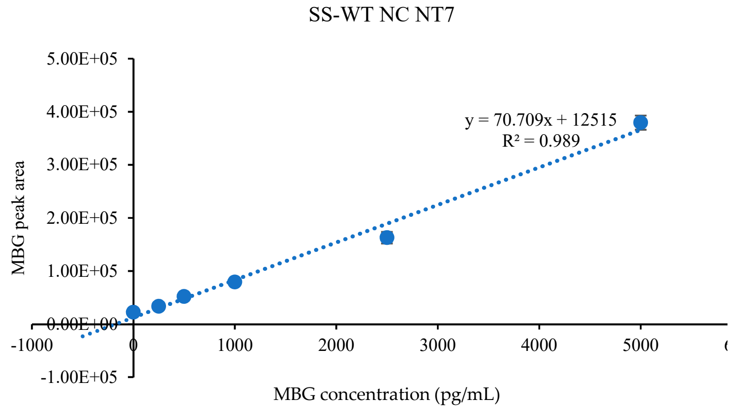
SS-WT NC NT7
y = 70.709x + 12515
R² = 0.989
MBG peak area
MBG concentration (pg/mL)

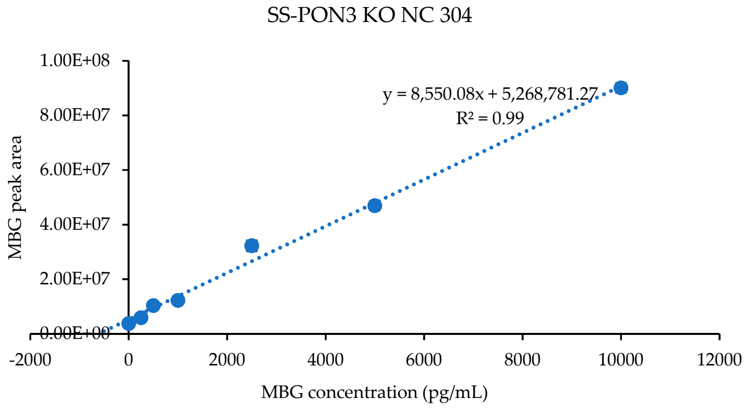
SS-PON3 KO NC 304
y = 8,550.08x + 5,268,781.27
R² = 0.99
MBG peak area
MBG concentration (pg/mL)

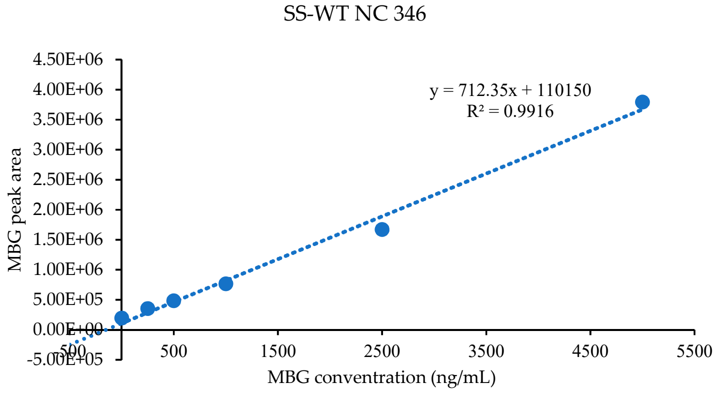
SS-WT NC 346
y = 712.35x + 110150
R² = 0.9916
MBG peak area
MBG conventration (ng/mL)

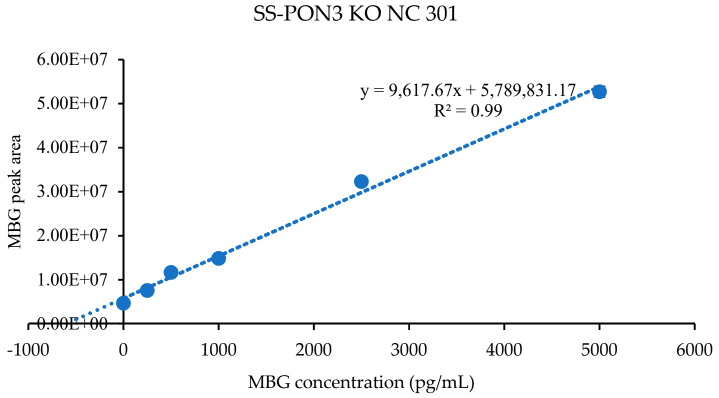
SS-PON3 KO NC 301
y = 9,617.67x + 5,789,831.17
R² = 0.99
MBG peak area
MBG concentration (pg/mL)

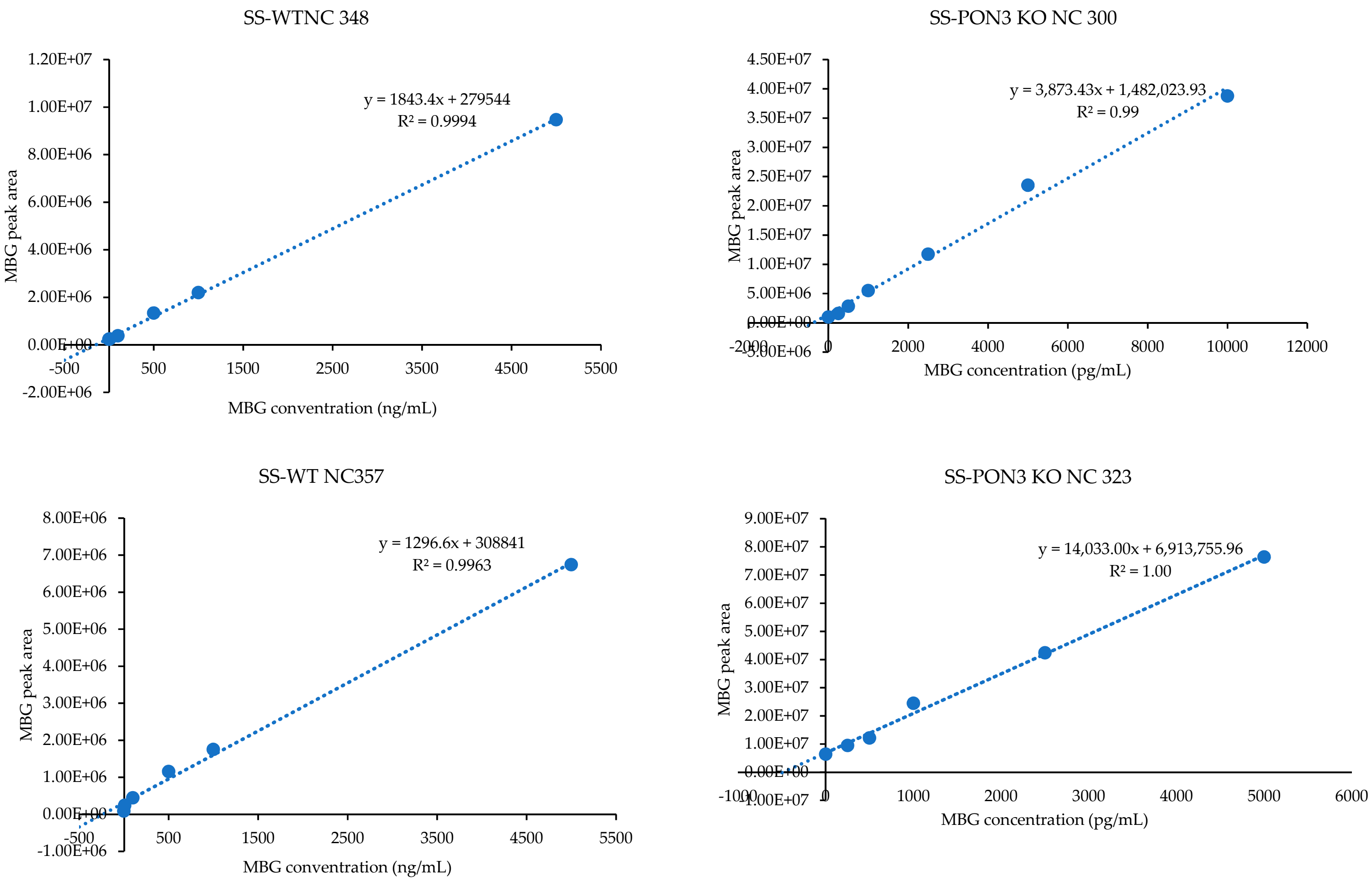

Figure S3. Standard addition calibration curves for quantification of MBG in SS-WT and SS-PON3 KO rat urine samples on a normal chow diet.

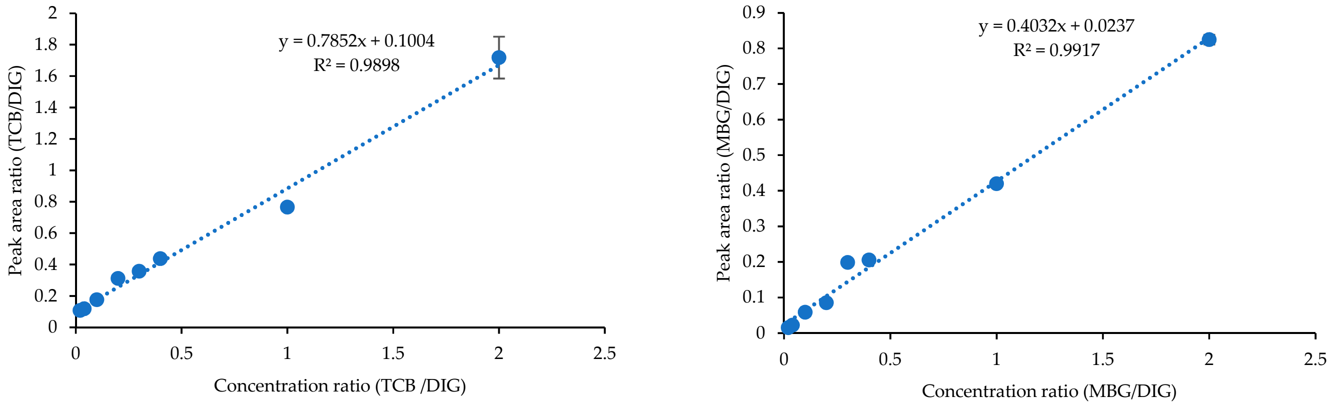

Figure S4. Internal standard calibration curves for quantification of TCB and MBG in SS-WT and SS- PON3 KO rat urine samples on a high salt diet.
